# Supplementary material for: COVID-19 Clinical Features and Outcome in Italian Patients Treated with Biological Drugs Targeting Type 2 Inflammation
Source: Life (Basel). 2024 Mar 13;14(3):378. doi: 10.3390/life14030378 (PMC10971220; doi:10.3390/life14030378)
Supplement: Supplementary file 1 [file life-14-00378-s001.zip › life-2890567-supplementary.pdf]

Questionnaire about COVID-19

Age: Gender:

Weight: High: BMI:

Smoking history:

Type 2 Disease:

- ☐ ASTHMA
- ☐ Chronic Rhinosinusitis with Polyposis (CRSWNP)
- ☐ Atopic dermatitis (AD)
- ☐ Chronic Spontaneous Urticaria (CSU)

Biological Therapy:

- ☐ No
- ☐ Yes

  If yes, specify ……………………………………………………………………………………….

Use of oral corticosteroids:

………

Other therapy:

………

Comorbidity:

………

SARS-CoV-2 vaccination at the time of infection:

- ☐ No one
- ☐ 1 Dose
- ☐ 2 Dose
- ☐ 3 Dose
- ☐ 4 Dose
- ☐ Other

Data of the positive swab for SARS-CoV-2:

Data of the negative swab for SARS-CoV-2:

Total Days of positivity:

Start day of Symptoms:

End day of Symptoms:

Total Days of Symptoms:

What symptoms did you experience during the infection? (check one or more…)

- ☐ None
- ☐ Asthenia
- ☐ Myalgia
- ☐ Anosmia and/or Ageusia
- ☐ Fever
- ☐ Common Cold symptoms
- ☐ Sore Throat
- ☐ Cough
- ☐ Pneumonia
- ☐ Dyspnoea
- ☐ Diarrhoea
- ☐ Other specifies

What therapy did you practice?

……....

Did you have an exacerbation of ASTHMA/CRSWNP/CSU/AD? If Yes, specify all the medication used.

………

Did you need hospitalization?

- ☐ No
- ☐ Yes

Complete only if your previous answer is Yes…

Date of hospitalization:

Date of hospital discharge:

Do you need Oxygen mask or any other support?

……

Thank you!
